# Assessing the Efficacy of Various Machine Learning Algorithms in Predicting Blood Pressure Using Pulse Transit Time

**DOI:** 10.3390/diagnostics15030261

**Published:** 2025-01-23

**Authors:** Ahmad F. Turki

**Affiliations:** 1 Electrical and Computer Engineering Department, Faculty of Engineering, King Abdulaziz University, Jeddah 21589, Saudi Arabia; aftorke@kau.edu.sa; 2 Center of Excellence in Intelligent Engineering Systems (CEIES), King Abdulaziz University, Jeddah 21589, Saudi Arabia

**Keywords:** pulse transit time (PTT), medical wearable devices, photoplethysmography (PPG), impedance plethysmography (IPG), electrocardiography (ECG), cuffless non-invasive blood pressure measurement, machine learning, AI prediction

## Abstract

**Background/Objectives:** This study investigates the potential of Pulse Transit Time (PTT) derived from Impedance Plethysmography (IPG), Photoplethysmography (PPG), and Electrocardiography (ECG) for non-invasive and cuffless blood pressure monitoring. IPG measures blood volume changes through electrical conductivity, while PPG detects variations in microvascular blood flow, providing essential insights for wearable health monitoring devices. **Methods:** Data were collected from 100 healthy participants under resting and post-exercise conditions using a custom IPG system synchronized with ECG, PPG, and blood pressure readings to create controlled blood pressure variations. Machine learning models, including Random Forest, Logistic Regression, Support Vector Classifier, and K-Neighbors, were applied to predict blood pressure categories based on PTT and cardiovascular features. **Results:** Among the various machine learning models evaluated, Random Forest demonstrated effective performance, achieving an overall accuracy of 90%. The model also exhibited robustness, effectively handling the challenge of unbalanced classes, with a 95% confidence interval (CI) for accuracy ranging from 80% to 95%. This indicates its reliability across different data splits despite the class imbalance. Notably, PTT derived from PPG emerged as a critical predictive feature, further enhancing the model’s ability to accurately classify blood pressure categories and solidifying its utility in non-invasive cardiovascular monitoring. **Conclusions:** The findings affirm the efficacy of using PTT measurements from PPG, IPG, and ECG as reliable predictors for non-invasive blood pressure monitoring. This study substantiates the integration of these techniques into wearable devices, offering a significant advancement for continuous, cuffless, and non-invasive blood pressure assessment.

## 1. Introduction

In recent years, the development of medical wearables has revolutionized preventive medicine by empowering individuals to monitor their health parameters continuously and non-invasively [1,2]. Among these innovations, non-invasive cuffless blood pressure monitoring systems (BPMSs) have emerged as a crucial advancement, providing an alternative to traditional sphygmomanometer methods [3]. The growing prevalence of hypertension—a major risk factor for cardiovascular diseases—underscores the need for wearable technologies that can integrate seamlessly into daily life to facilitate routine monitoring [4].

While the last decade has seen significant growth in wearable health devices, such as fitness trackers and ECG monitors, the potential of these devices for accurate blood pressure monitoring is yet to be fully realized [5]. Traditional blood pressure (BP) monitors often require the use of a cuff, which can be cumbersome and uncomfortable, thus limiting their integration into wearable formats [6]. Recent advancements in artificial intelligence (AI) and machine learning have begun to address these limitations by enhancing the accuracy and reliability of cuffless BP monitoring methods [7]. These technologies leverage data from sensors, employing algorithms that improve over time to offer more precise measurements without the need for bulky or invasive equipment.

Particularly, the use of Pulse Transit Time (PTT) measurements, which require sophisticated algorithms to accurately estimate blood pressure from sensor data, represents a significant area of research [8]. The integration of AI helps refine these measurements, providing a foundation for continuous and non-invasive monitoring solutions [8]. Studies like those by Carek, Andrew et al., and Gesche et al. have shown that devices based on PTT can offer comparable accuracy to traditional methods, suggesting a viable path forward for their use in everyday health monitoring [9,10].

Building on this foundation, our research focuses on further enhancing the reliability of these technologies through advanced machine learning models. We explore various algorithms’ effectiveness in interpreting data from Impedance Plethysmography (IPG) and Photoplethysmography (PPG), two prominent methods for capturing cardiovascular dynamics. This study not only aims to improve the accuracy of BP measurements but also to expand the functionality of wearable devices, making them more adaptable to the needs of individuals seeking to manage their cardiovascular health proactively.

## 2. Related Work

This section explores recent advancements and challenges in cuffless blood pressure (BP) monitoring, focusing on calibration stability, the reliability of Pulse Transit Time (PTT) and pulse arrival time (PAT), and validation issues for wearable devices. It also reviews studies integrating machine learning and artificial intelligence to enhance BP prediction, highlighting their potential and limitations.

### 2.1. Challenges and Advances in Cuffless Blood Pressure Monitoring

In the field of cuffless blood pressure (BP) monitoring, several studies underscore the complexities and limitations inherent in this non-invasive technology, particularly when used in clinical settings.

The study “Assessment of Calibration Models for Cuff-Less Blood Pressure Measurement After One Year of Aging” by Mohammad Yavarimanesh and colleagues evaluated the durability of calibration models in cuffless blood pressure (BP) monitors over one year [11]. It was found that these models, except for one based on toe pulse arrival time (PAT), required frequent recalibration due to changes associated with vascular aging [11]. The study involved multiple physiological tests to assess the accuracy of these models using pulse waveforms from different body locations [11]. The toe PAT model proved to be the most stable, suggesting it might only need annual recalibration [11]. This highlights a significant limitation of cuffless BP monitors, namely, the need for regular recalibration to maintain accuracy, which could impact user adherence and the practicality of long-term monitoring.

The study by Guanqun Zhang and colleagues critically examined the efficacy of pulse arrival time (PAT) compared to Pulse Transit Time (PTT) as indicators of blood pressure (BP) [12]. Despite the simplicity of measuring PAT, which includes the pre-ejection period along with PTT, the study showed that PAT is not a reliable surrogate for PTT in tracking blood pressure changes [12]. Through experiments on dogs subjected to a variety of hemodynamic interventions to induce wide BP changes, it was demonstrated that PAT, because of its inclusion of the pre-ejection period, does not correlate as closely with BP changes as PTT [12]. The results show that while PTT had a strong inverse correlation with BP, providing a reliable measure after calibration, PAT did not maintain this reliability and often failed to meet the standard BP error limits set by the FDA [12]. The study concluded that PAT’s additional complexity, influenced by cardiac dynamics directly unrelated to arterial pulse transit, limits its utility as a standalone measure for non-invasive and cuffless BP monitoring [12].

Mukkamala et al. critically evaluated the accuracy of cuffless blood pressure measurement devices, emphasizing the unresolved challenges these technologies face despite their potential for transforming hypertension management [13]. The authors highlighted the significant gap between the theoretical promise of cuffless devices and their practical performance, notably their failure to meet the validation standards set by the 2018 Universal Standard for automated blood pressure devices developed by the AAMI/ESH/ISO [13]. The study underscores that current validation protocols are ill-suited for cuffless technologies, necessitating novel approaches to assess their accuracy and reliability [13]. Furthermore, Mukkamala and colleagues stressed that the European Society of Hypertension (ESH) Guidelines from 2021 do not recommend cuffless devices for clinical use due to these unresolved accuracy and validation issues [13]. The review serves as a crucial reminder of the meticulous scrutiny required when evaluating emerging medical technologies to ensure they deliver their intended benefits without misleading conclusions driven by inadequate methodologies [13].

In contrast, our study targets everyday use by healthy individuals, focusing on wearable devices and machine learning models for non-invasive BP classification. While Mukkamala et al. prioritized clinical-grade accuracy, our research aims to enhance usability and classification performance in real-world and non-clinical settings.

Gogiberidze et al. introduced and evaluated a novel cuffless blood pressure measurement method integrated within a smartphone case, known as the CardioQVARK device (CardioKVARK, Moscow, Russia) [14]. Their study, involving 167 patients with varying blood pressure levels, assessed the device’s accuracy against traditional cuff-based mercury sphygmomanometers [14]. The findings revealed a mean absolute difference for systolic and diastolic blood pressure within acceptable limits according to IEEE standards [14]. Notably, the device demonstrated particularly effective performance in patients with prehypertension, with smaller measurement errors compared to those with higher hypertension stages [14]. While the device complied with IEEE standards, the study acknowledged potential biases, especially in measuring diastolic blood pressure, suggesting limited suitability for screening but confirming utility for ongoing monitoring [14]. These results underscore the evolving landscape of cuffless devices in blood pressure monitoring, emphasizing the need for meticulous validation to ensure clinical reliability and epidemiological relevance [14].

In comparison, our study emphasizes everyday use for healthy individuals, leveraging wearable devices and machine learning for BP classification. While Gogiberidze’s work was aimed at clinical applications and rigorous validation protocols, our focus lies in enhancing accessibility and convenience for general health tracking. Additionally, while their study population consisted predominantly of hypertensive patients, our research targets healthy users to support non-clinical and real-world preventive care. Thus, while Gogiberidze et al. prioritized clinical accuracy, our work advocates for user-friendly and non-invasive technology to promote health awareness and early intervention in a broader population.

Overall, while cuffless BP devices present a non-intrusive and continuous monitoring advantage, their current limitations necessitate cautious interpretation of data and significant technological and clinical advancements to ensure their reliability and accuracy in healthcare settings. The pursuit of enhanced cuffless BP monitoring technologies must continue to address these challenges comprehensively, aiming to bridge the gap between current invasive methods and potential future non-invasive solutions.

### 2.2. Current Trends in Cuffless Blood Pressure Monitoring

The article “Cuffless blood pressure measuring devices: review and statement by the European Society of Hypertension Working Group on Blood Pressure Monitoring and Cardiovascular Variability” by George S. Stergiou et al. provides an extensive review of the current landscape of cuffless BP measuring technologies [15]. Despite the potential of these devices to enhance hypertension management by providing continuous non-invasive measurements, the European Society of Hypertension does not recommend their use for the diagnosis or management of hypertension [15]. Therefore, our study emphasizes usability, accessibility, and reliable classification accuracy tailored specifically to healthy individuals in non-clinical everyday settings.

The large-scale validation study on cuffless blood pressure (BP) measurement using smartwatches by Liu et al. presents distinct approaches and outcomes in developing non-invasive BP monitoring solutions [16]. Liu et al.’s study leveraged a large cohort (3077 participants) and evaluated various machine learning (ML) and deep learning (DL) algorithms, including calibration-based and calibration-free models, across a broad population [16]. The best-performing calibration-based models achieved mean estimation errors of 1.33 ± 6.43 mmHg for diastolic BP (DBP) and 2.31 ± 9.57 mmHg for systolic BP (SBP), with high reliability for normotensive and younger participants but reduced accuracy for hypertensive and older individuals [16]. In contrast, the calibration-free models displayed significantly lower performance, highlighting the critical role of individual calibration in accurate BP estimation [16].

Alternatively, our study differs in focus and methodology, emphasizing usability, accessibility, and acceptable classification accuracy for healthy users in non-clinical environments. Additionally, our approach integrates advanced signal processing techniques and user-friendly designs, enabling efficient BP monitoring without frequent recalibration.

While both studies highlight the potential of wearable technologies for BP monitoring, Liu et al.’s work underscores the challenges of scalability and accuracy across diverse populations, particularly in hypertensive and elderly subgroups [16].

Matsumura et al. introduced a cuffless method for estimating blood pressure (BP) using heart rate (HR) and modified normalized pulse volume (mNPV) obtained via a smartphone application [17]. Their simplified approach achieved a correlation coefficient exceeding 0.70 for mean arterial pressure (MAP), systolic BP (SBP), and diastolic BP (DBP) compared to standard brachial cuff measurements, demonstrating the potential for accessible and portable BP monitoring [17]. This method’s strength lies in its reliance on minimal hardware and the absence of calibration or additional sensors, making it highly user-friendly [17]. However, it was tested on a limited cohort of young healthy participants under controlled conditions, which restricts its generalizability to broader populations [17]. Conversely, our study integrates multi-signal data from Impedance Plethysmography (IPG), Photoplethysmography (PPG), and Electrocardiography (ECG) to enhance predictive accuracy. By leveraging advanced machine learning models such as Random Forest, achieving 90% accuracy with an AUC of 0.96, our approach aims to bridge the gap between simplicity and reliability. While Matsumura et al.’s work emphasized accessibility, our research targets robustness and adaptability across diverse conditions, expanding the potential applications of non-invasive BP monitoring technologies for everyday use.

### 2.3. Advancements and Challenges in Machine Learning Applications for Hypertension Management

In the study titled “Machine Learning in Hypertension Detection: A Study on World Hypertension Day Data”, supervised ML algorithms were applied to a substantial dataset collected in Italy by the SIIA (Italian Society of Arterial Hypertension) [18]. While these algorithms were able to identify hypertension with modest accuracy and suboptimal sensitivity, the study suggests that with further refinement and testing across diverse populations, such ML approaches could become valuable for hypertension screening [18]. This could potentially provide a cost-effective alternative to traditional physician evaluations [18]. The study advocates for future research to focus on developing clinical models that not only enhance diagnostic accuracy but also reduce costs, particularly in resource-limited settings [18].

Building on this foundation, the study “Predicting Hypertension Control Using Machine Learning” marks a significant advancement by being the first to use ML for predicting blood pressure control [19]. The study demonstrated that ML models could predict blood pressure control within 12 months of a patient encounter with reasonably high accuracy [19]. The results emphasize the potential of ML to shorten the time required to achieve blood pressure control, provided that the models are validated and improved through further research [19]. This highlights an exciting direction for ML in enhancing patient outcomes through timely intervention [20].

However, integrating machine learning into hypertension management presents several challenges. The research titled “AI, Machine Learning, and ChatGPT in Hypertension” addressed some of these limitations [21]. It underscores the critical role of data quality in developing effective ML models [21]. The study points out that a significant portion of resources in data science projects is devoted to data collection, cleaning, and validation [21]. Issues such as erroneous data points and compatibility problems between different coding systems can impair model performance [21]. Thus, ensuring high-quality data is crucial for the success of ML in medical applications, requiring careful attention to both intuitive and less obvious data errors [21].

Furthermore, the study “Future Directions for Using Artificial Intelligence to Predict and Manage Hypertension” outlined a vision for leveraging AI to advance our understanding of hypertension [22]. This includes exploring epigenetic changes, early-stage physiological indicators, and personalized treatment approaches based on a multi-omic perspective [22]. AI holds the promise of not only identifying individual risk factors and mechanisms for poorly controlled hypertension but also of standardizing BP measurements and improving their integration into clinical practice [23]. Leading software companies and academic institutions are already engaging in AI research, but there is a call for more evidence, particularly in deep learning (DL) applications and mobile health interventions, to validate these technologies effectively [22].

## 3. Materials and Methods

### 3.1. Construction of the Impedance Plethysmography System

A custom-built circuit containing an instrument amplifier, a demodulation circuit, and a bandpass filter was developed to amplify and clarify the impedance signal captured from the armband electrodes.

**Figure 1:**
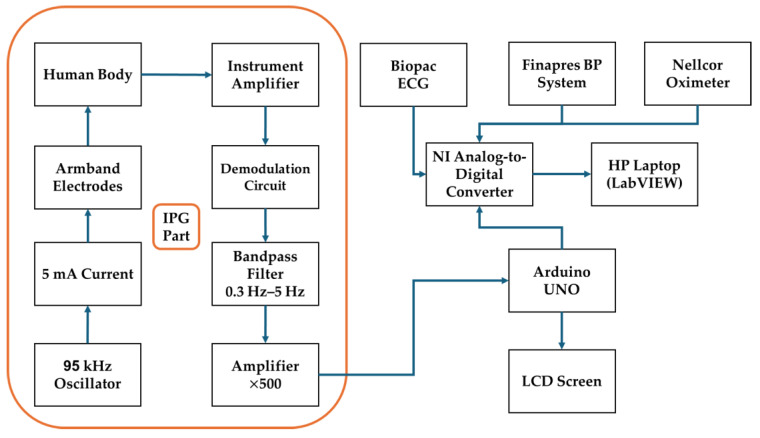
System architecture for Impedance Plethysmography-based non-invasive blood pressure monitoring with integrated sensors and data acquisition components.

Figure 1 presents a streamlined design of the Impedance Plethysmography (IPG) system used in this study, illustrating the flow of components from signal acquisition to display. The IPG part includes armband electrodes placed on the body, which receive a stable 5 mA current generated by a 95 kHz oscillator. The resulting impedance changes, indicative of blood volume variations, are amplified and processed through an instrument amplifier, a demodulation circuit, and a bandpass filter. This filtered signal is then further amplified for clarity. The processed data are sent to an Arduino UNO microcontroller.

Constructing this IPG system was the first essential step in this study, enabling the accurate and non-invasive measurement of cardiovascular parameters required for further analysis and model assessment. By integrating the IPG signal with the ECG signal, the impedance and ECG data were leveraged. By capturing both cardiovascular impedance changes and ECG timing, this setup can provide a non-invasive solution to continuous blood pressure tracking.

#### IPG Component

The IPG part (outlined in orange) includes several key elements that facilitate the detection of blood volume changes:

Human body interface: Armband electrodes are placed on the body to measure impedance changes related to blood flow.

Oscillator (95 kHz): A 95 kHz oscillator generates a high-frequency current, which is applied to the body through the electrodes. This frequency is selected to minimize interference from other physiological signals and to optimize sensitivity to impedance changes.

5 mA current source: A constant 5 mA current is applied via the armband electrodes to measure impedance changes due to varying blood volume within the body segment.

Signal amplification: The signal is first processed by an instrument amplifier to enhance weak impedance signals, followed by a demodulation circuit to extract relevant information.

Bandpass filtering: The bandpass filter employed in our study is a hardware-based implementation integrated within the custom-made circuit. It is designed to isolate the frequency range of 0.3 Hz to 5 Hz, which is critical for capturing cardiovascular dynamics. This range effectively excludes high-frequency noise and very low-frequency drift, which are not relevant to the physiological signals we intended to analyze. The filter’s hardware integration ensures minimal delay and high fidelity in real-time signal processing during data acquisition from the electrodes connected to the arm.

High-gain amplifier (×500): The filtered signal is further amplified to improve signal clarity, making it suitable for subsequent processing and analysis.

Arduino UNO: an Arduino UNO (Arduino SRL, Turin, Italy) microcontroller processes the IPG signals. It also communicates with the LCD screen.

LCD screen: It displays the status of the electrode connectivity.

**Figure 2:**
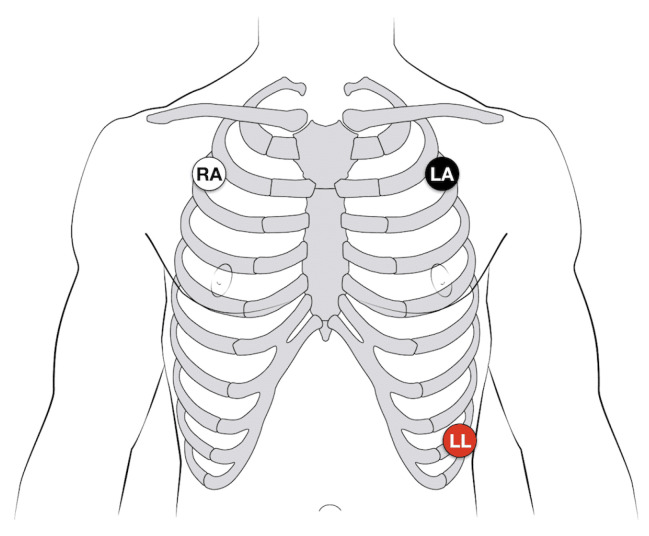
ECG chest 3-lead electrode placement, RA (Right Arm): Electrode on right arm for reference and circuit completion, LA (Left Arm): Electrode on left arm captures lateral heart signals, LL (Left Leg): Electrode on left leg acts as ground, reducing noise [24].

Using a 3-electrode configuration, the ECG electrodes are placed strategically on the body, as shown in Figure 2.

### 3.2. Data Collection for System Comparison and Validation

To comprehensively study the relationship between Impedance Plethysmography (IPG) Pulse Transit Time (PTT) and blood pressure, with the aim of assessing the feasibility of predicting blood pressure from IPG PTT, data collection was conducted under two distinct physiological conditions: at rest and post-exercise. A rest mode of 5 min was established initially to obtain baseline measurements, after which subjects engaged in physical exercise until their heart rate increased by 20% above the resting rate. This increase was targeted to induce physiological changes in blood pressure, creating a controlled variation in cardiovascular response that facilitates a robust comparison of PTT across different blood pressure states. Following the exercise-induced increase, measurements were taken for an additional 5 min to capture the immediate post-exercise phase.

Data from the IPG system were collected concurrently with signals from the BIOPAC MP150 ECG system (BIOPAC Systems, Goleta, CA, USA), the Finapres^®^ NOVA hemodynamic monitoring system (Finapres Medical Systems, Enschede, The Netherlands), and a Photoplethysmography (PPG) signal obtained from the Nellcor N600x Pulse Oximeter (Medtronic, Dublin, Ireland). This multi-system setup enabled a comprehensive analysis of PTT variability in relation to fluctuating blood pressure values, providing insights into the potential of IPG PTT as a non-invasive predictor of blood pressure.

A total of 100 healthy individuals participated in this study, representing a balanced sample of the adult population, composed of 50 males and 50 females. The average age of the participants was 24.2 ± 6.34 years, and the average BMI was 27.5 ± 4.6, reflecting data for the entire group. Participants were included in this study if they were adults aged 18–35, without any known chronic diseases. The exclusion criteria were specifically designed to omit individuals with any health conditions, such as hypertension, diabetes, or chronic pulmonary diseases, or those on medications that could affect blood pressure or oxygen saturation, such as beta-blockers, diuretics, or corticosteroids.

Measurements were collected before and after exercise for each participant, capturing data from the IPG system, BIOPAC ECG, the Nellcor oximeter, and the Finapres^®^ NOVA system. Each device recorded analog signals at a sampling rate of 256 Hz, which were digitized using a National Instruments NI-USB-6128 analog-to-digital converter and stored on an HP ProBook laptop. Data acquisition was managed using LabVIEW 2024 (National Instruments, Austin, TX, USA), with subsequent offline analysis conducted in MATLAB R2022b (MathWorks, Natick, MA, USA).

**Figure 3:**
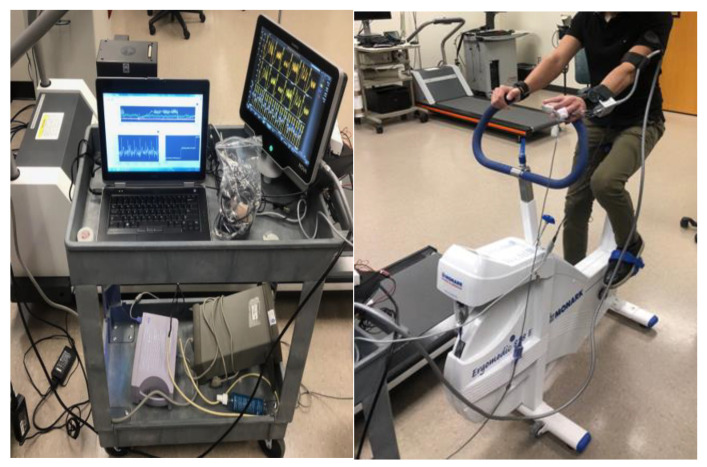
Data collection setup.

The exercise component involved participants using the Monark 828E Ergomedic Microfit Robobike (Monark Exercise AB, Vansbro, Sweden) as shown in Figure 3, with measurements taken both at rest and after a controlled physical exertion designed to increase heart rate by approximately 20% above resting levels. The workload was adjusted to consistently achieve this heart rate increase, simulating a mild cardiovascular load and allowing for dynamic blood pressure responses.

This study adhered to ethical standards, with approval from the Institutional Review Board (IRB) of the Center of Excellence in Intelligent Engineering Systems at King Abdulaziz University (approval number 22-CEIES-Biomed-2024). All participants provided informed consent, ensuring compliance with ethical guidelines.

### 3.3. Understanding Blood Pressure and Related Cardiovascular Measurements

For classification purposes, blood pressure levels were organized into categories, including normal, elevated, and hypertensive stages, in accordance with established clinical guidelines. These categories allowed us to systematically analyze and predict blood pressure status, assessing the efficacy of machine learning algorithms in identifying and categorizing these distinct blood pressure ranges.

To clarify the categorization of blood pressure levels used in our study, Table 1 presents the definitions of healthy and unhealthy blood pressure ranges. These categories align with the 2017 guidelines from the American Heart Association; however, it is important to note that the updated 2023 guidelines were not considered in this study [25].

In addition to standard blood pressure readings, this study incorporated several supplementary cardiovascular measurements to provide a more comprehensive view of cardiovascular health. Heart rate (HR), measured as the number of heart beats per minute, served as a key parameter reflecting cardiac function. Two types of PTT were also used: PTT from Photoplethysmography, which captures the time it takes for the pulse wave to travel between two points using optical methods, and PTT from Impedance Plethysmography (PTT IPG), which measures the pulse transit through impedance changes. Both PTT PPG and PTT IPG are important indicators for estimating blood pressure, with PTT IPG providing an alternative approach to PTT PPG for more nuanced readings. By incorporating these parameters, we aimed to enhance the predictive capabilities of this study’s models and to better understand how these physiological signals relate to blood pressure changes.

Furthermore, Pulse Wave Velocity (PWV) values from both Photoplethysmography (PWV PPG) and Impedance Plethysmography (PWV IPG) were used as critical features to assess the performance of machine learning models in predicting blood pressure categories. PWV, being a key indicator of arterial stiffness, provided valuable information on cardiovascular health, allowing the models to better classify blood pressure status based on physiological characteristics. Table 2 provides a comprehensive summary of the demographic data and physiological variables measured both before and after exercise.

### 3.4. Assessing Machine Learning Algorithms for Blood Pressure Prediction Using PTT

The collected data were processed using various machine learning classification algorithms to assess the efficacy of predicting blood pressure categories based on PTT measurements and the other cardiovascular parameters. The analysis primarily focused on two outputs for each algorithm: (1) the overall prediction accuracy, as indicated by performance metrics such as accuracy, precision, recall, and F1-score, and (2) feature importance analysis using SHAP (SHapley Additive exPlanations) values to interpret the impact of each feature on the model’s predictions. The analysis was conducted in Python version 3.11.5 (Python Software Foundation, Wilmington, DE, USA).

The dataset consisted of all previously mentioned features, including systolic and diastolic blood pressure values. These features were selected as predictive inputs, with the target variable representing blood pressure status, categorized as either normal or elevated. A supervised machine learning approach was applied in this analysis as labeled data were available to train the model on the relationship between input features and blood pressure categories.

The data were split into training and testing sets, with 70% of the data used for training and 30% reserved for testing, ensuring an unbiased model evaluation. Standard scaling and Min-Max scaling were applied to normalize feature distributions based on the specific requirements of each model, enhancing the model’s performance and accuracy in predicting blood pressure status.

To evaluate the efficacy of different machine learning models for predicting blood pressure status, we tested four classification algorithms: Random Forest, Logistic Regression, Support Vector Classifier (SVC), K-Neighbors Classifier (KNN), and Naïve Bayes.

The Random Forest classifier, an ensemble model, was configured with 150 estimators, a maximum depth of 10, and a minimum sample split of 5. Standard scaling was applied to this model to ensure consistent feature scaling across the trees, enhancing the stability of the model’s predictions.

The Logistic Regression model was configured as a linear classifier, was trained with a maximum iteration limit of 1000 to support model convergence, and used a regularization parameter C=0.5 to prevent overfitting. This model employed the ‘liblinear’ solver, which is suitable for binary classification problems, and Min-Max scaling was applied to normalize feature values between 0 and 1, improving model performance by aligning the feature range.

The Support Vector Classifier (SVC) was configured with a linear kernel to create a clear decision boundary, a degree of 4, gamma set to ‘auto’ to automatically scale based on feature variance, and a regularization parameter C = 1.0 to balance margin maximization with error minimization. This model was also standardized using Standard scaling, which ensured data centering and scaling, facilitating robust model performance and enabling probability estimates for improved interpretability.

The K-Neighbors Classifier (KNN) was configured with 5 neighbors, using ‘distance’ weighting to assign more influence to closer points in classification decisions. Min-Max scaling was applied to normalize the feature range, ensuring that the distance-based algorithm could more accurately assess feature relationships across the scaled dataset.

Finally, the Naïve Bayes classifier was implemented using Gaussian Naïve Bayes (GaussianNB), which assumes that continuous features, such as HR, PTT, and PWV, follow a Gaussian distribution. This probabilistic approach is effective for handling clinical datasets with continuous variables and imbalanced classes. Using Stratified K-Fold cross-validation with five splits ensured balanced representation across blood pressure categories. Key metrics, including precision, recall, F1-score, accuracy, and AUC, were calculated for each fold, with 95% confidence intervals to evaluate reliability. GaussianNB efficiently leveraged feature relationships for classification, making it suitable for the dataset’s continuous and variable nature.

## 4. Data Analysis

**Figure 4:**
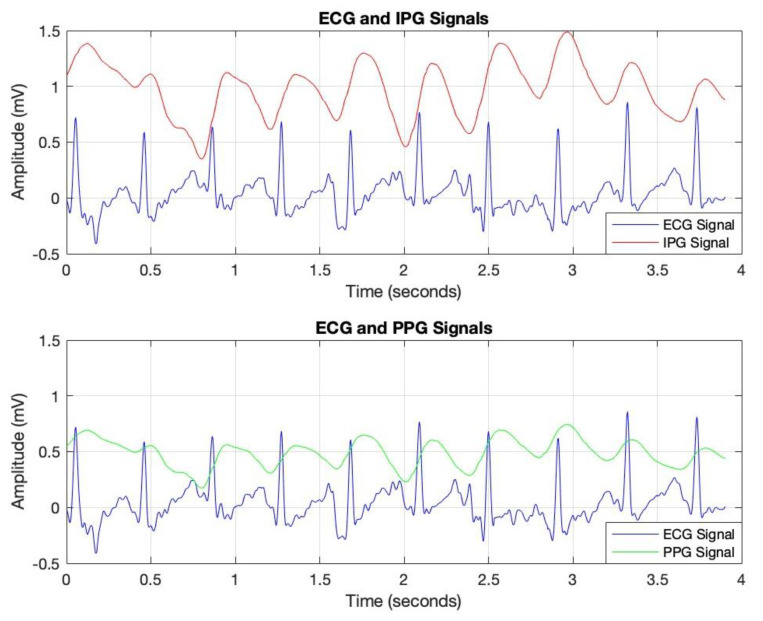
Comparison of ECG signals with IPG and PPG signals for Pulse Transit Time (PTT) measurements.

The data analysis commenced with the processing of IPG, ECG, and PPG signals, sampled at a rate of 256 Hz, as depicted in Figure 4. This sampling rate was selected to ensure accurate calculation of Pulse Transit Time (PTT), as outlined in the methodology section. Data acquisition was conducted under both resting and post-exercise conditions to introduce controlled variability in cardiovascular responses, thereby enhancing the robustness of the analysis.

**Figure 5:**
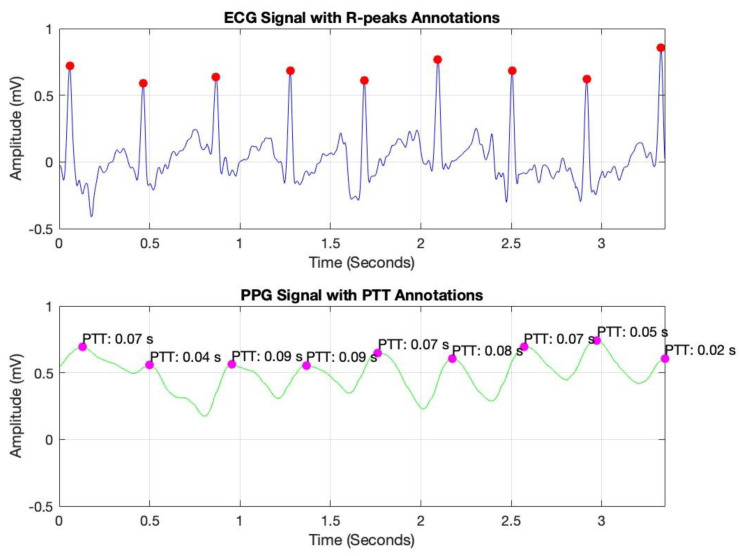
ECG and PPG signals with detected peaks and Pulse Transit Time (PTT) annotations.

To optimize signal quality, a bandpass filter was applied to each signal, isolating the relevant frequency ranges associated with cardiovascular dynamics. This filtering process effectively attenuated noise and extraneous frequencies, thereby improving the clarity and reliability of the data for subsequent PTT calculations. In Figure 5, peak detection was conducted using MATLAB’s findpeaks command, identifying R-peaks in the ECG signal (marked with red dots) and corresponding peaks in the PPG signal (marked with magenta dots). By measuring the time interval between these peaks, we derived the PTT values, which are essential for non-invasive blood pressure estimation.

Pulse Transit Time (PTT) is typically calculated as the time difference between the R-peak in the ECG signal (indicating the onset of the cardiac cycle) and the corresponding peak in a peripheral signal, such as the PPG or IPG signal (indicating the arrival of the pulse wave at a distal site) [10]. The formula for PTT can be expressed as [10]:

*PTT = t*_PPG/IPG_ − *t*_ECG_ (1)

where *t*_PPG/IPG_ is the time of the peak in the PPG or IPG signal, marking the arrival of the pulse wave at the peripheral measurement site.

*t*_ECG_ is the time of the R-peak in the ECG signal, representing the start of the cardiac cycle.

These annotated plots validate the system’s accuracy in capturing key cardiovascular parameters, supporting the reliability of PTT as a foundation for continuous blood pressure monitoring.

Furthermore, the heart rate (HR) calculation was based on the *R-R interval*, which represents the time between consecutive R-peaks in the ECG signal.

Given a sampling rate of 256 Hz, the *R-R interval* was determined by measuring the difference between the indices (*i*) of consecutive R-peaks and then dividing by the sampling rate to convert this value into seconds, as follows:

(2) $$R-R\;interval=\frac{\mathrm{peak}\_\mathrm{locs}(i+1)-\mathrm{peak}\_\mathrm{locs}(i)}{sampling\;rate}$$

The HR in beats per minute was then calculated as the reciprocal of the R-R interval, multiplied by 60:

(3) $$HR=\frac{60}{R-R\;interval}$$

As previously discussed, the performance of the machine learning models was evaluated using key metrics, namely, accuracy, precision, recall, and F1-score, as outlined below.

Accuracy: This metric assesses the overall correctness of the model by calculating the ratio of correctly classified instances (both true positives and true negatives) to the total number of instances. It provides a general measure of the model’s effectiveness in making accurate predictions across all classes [26].

(4) $$Accuracy=\frac{\left(TP+TN\right)}{\left(TP+TN+FP+FN\right)}\times 100$$

Precision: This metric emphasizes the accuracy of the model’s positive predictions by calculating the proportion of true positives among all instances predicted as positive. Precision reflects the model’s ability to avoid false positives, indicating how reliable its positive predictions are [26].

(5) $$Precision=\frac{TP}{\left(TP+FP\right)}\times 100$$

Recall (or sensitivity): This metric evaluates the model’s ability to identify all relevant instances in the dataset. It is calculated as the proportion of true positives out of all actual positive instances, indicating how effectively the model captures the true positives and minimizes false negatives [26].

(6) $$Recall=\frac{TP}{\left(TP+FN\right)}\times 100$$

where for Equations (2)–(4), TP: True Positive, TN: True Negative, FP: False Positive, and FN: False Negative.

F1-score: This metric provides a balanced evaluation of a model’s performance across individual classes, offering a more nuanced assessment than overall accuracy alone. The F1-score combines precision and recall, calculating their harmonic mean to deliver a single metric that accounts for both false positives and false negatives. This makes it particularly useful for models where balancing precision and recall is essential, providing a comprehensive measure of the model’s predictive ability [26,27].

(7) $$\mathrm{F1-Score}=2\times \frac{Precision\times Recall}{\left(Precision+Recall\right)}\times 100$$

Additionally, feature importance analysis was conducted using SHAP (SHapley Additive exPlanations) to interpret the contributions of each feature to the machine learning model’s predictions. SHAP values quantify the impact of each feature on the final prediction, highlighting both the individual influence of each feature and its relative importance compared to others. This approach also reveals how interactions between features affect model outcomes, providing a comprehensive understanding of the model’s reliance on specific input variables [28].

(8) $$\phi \left(f\right)=\phi_{0}+\sum_{j=1}^{M}\frac{f\left(x_{i}\right)-E\left[f\left(x_{i}\right)\right]}{M}$$

where:

*ϕ*(*f*) represents the SHAP values for a particular feature.

*ϕ*_0_ is the expected value of the model’s prediction.

*M* is the total number of features.

*f*(*x_i_*) is the model’s prediction when feature *i* is included.

*E*[*f*(*x_i_*)] is the expected prediction when feature *i* is excluded.

## 5. Results

This section presents the performance of various machine learning models for blood pressure classification, their metrics, such as accuracy, precision, recall, and F1-score, and their confidence intervals. Additionally, key features influencing model performance are highlighted using SHAP analysis.

### 5.1. Demographic and Physiological Variables

Table 2 summarizes the demographic details and physiological measurements of the study participants, highlighting significant changes in key metrics such as heart rate, Pulse Transit Time, and blood pressure before and after exercise.

### 5.2. Model Performance Metrics

The performance metrics of the machine learning models, including accuracy, precision, recall, and F1-score, are presented in Table 3. These metrics evaluate the ability of each model to classify blood pressure categories effectively, with both macro and weighted averages provided for a balanced assessment.

### 5.3. Confidence Intervals of Metrics

To assess the robustness of each model, confidence intervals for key metrics, including precision, recall, F1-score, accuracy, and AUC, are summarized in Table 4.

### 5.4. AUC by Blood Pressure Class

Table 5 outlines the AUC values for each blood pressure class across all models, providing insights into the classification performance for specific BP categories.

### 5.5. Model-Specific Analysis

#### 5.5.1. Random Forest

**Figure 6:**
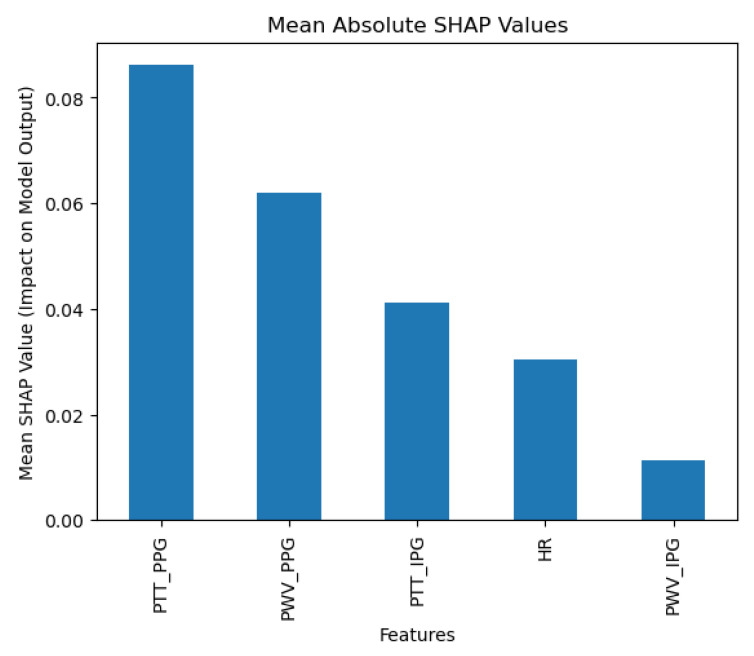
Random Forest SHAP results.

The Random Forest classification algorithm exhibited exceptional predictive performance, achieving an accuracy of 0.90, with a 95% confidence interval of 0.80–0.95. The model reported a macro average precision of 0.95, recall of 0.81, and F1-score of 0.82, indicating a strong overall ability to identify each category of blood pressure accurately, despite varying sample sizes per class. The weighted averages further underscore its efficacy: precision of 0.92 (95% CI: 0.80–0.98), recall of 0.90 (95% CI: 0.81–0.95), and F1-score of 0.88 (95% CI: 0.77–0.95). These metrics reveal that the model not only accurately classifies the majority of the class but does so with considerable balance across different categories, effectively adjusting for class imbalances. SHAP analysis (Figure 6) reveals that the Random Forest model heavily relies on Pulse Transit Time from Photoplethysmography (PPT_PPG) as a key feature, underscoring its importance in the prediction of blood pressure categories.

#### 5.5.2. Logistic Regression

The Logistic Regression model yielded an accuracy of 0.70, with confidence intervals spanning from 0.72 to 0.84, indicating moderate reliability in its predictions. The macro averages for precision, recall, and F1-score were 0.59, 0.54, and 0.47, respectively. These lower macro averages suggest that the model’s performance is not uniform across all classes, likely reflecting challenges in handling class imbalances or distinguishing between more nuanced differences in the blood pressure categories.

On the other hand, the weighted averages—precision of 0.67, recall of 0.70, and F1-score of 0.60—indicate slightly better performance when accounting for the prevalence of each class. The weighted metrics, which were higher than the macro averages, suggest that the model performs better on more common classes but struggles with less frequent categories.

**Figure 7:**
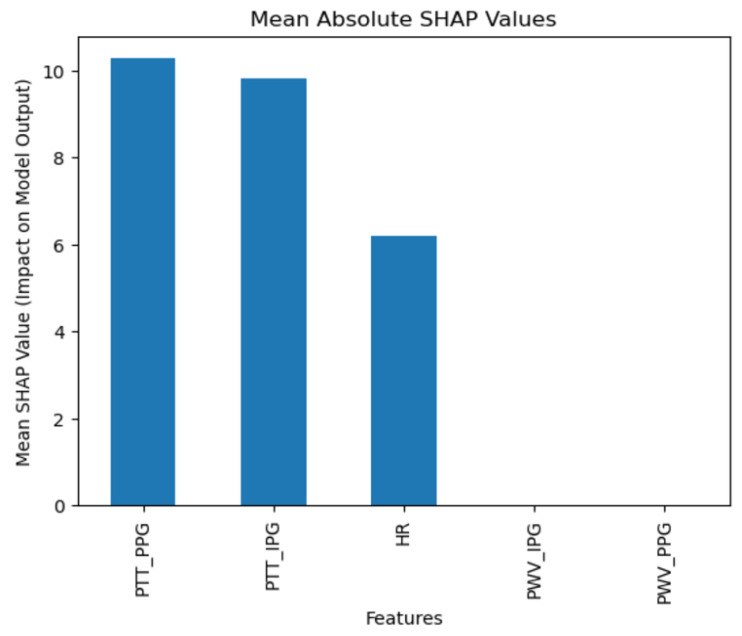
Logistic Regression SHAP results.

SHAP analysis (Figure 7) highlights Pulse Transit Time from Photoplethysmography (PPT_PPG) as a critical feature, similar to Random Forest, indicating its role in blood pressure prediction.

#### 5.5.3. Support Vector Machine (SVM)

The SVM model achieved an accuracy of 0.83, with its confidence intervals for accuracy ranging from 0.71 to 0.89. This indicates a relatively high level of consistency in SVM’s predictions across different subsets of data. The macro averages for precision, recall, and F1-score were 0.66, 0.71, and 0.68, respectively, suggesting that SVM performs reasonably well across all categories but with some room for improvement, especially in precision and F1-score.

The weighted averages—precision of 0.74, recall of 0.83, and F1-score of 0.78—indicate that the model is more effective when adjusted for the prevalence of each class. These metrics suggest that SVM is particularly adept at handling the dominant classes in the dataset but may not perform as well on minority classes without further adjustments or tuning.

**Figure 8:**
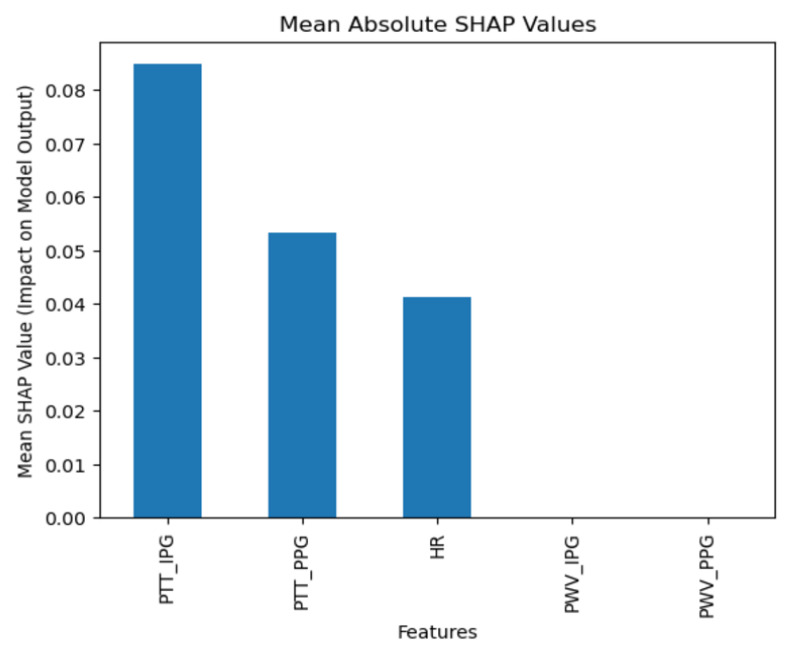
Support Vector Machine SHAP results.

SHAP analysis (Figure 8) for SVM highlights the importance of Pulse Transit Time from Impedance Plethysmography (PPT_IPG), suggesting this feature’s relevance in supporting model performance.

#### 5.5.4. K-Nearest Neighbors (KNN)

The KNN model displayed a high level of accuracy at 0.93, with confidence intervals ranging from 0.63 to 0.87. This wide range suggests some variability in KNN’s performance across different data subsets or potential overfitting to the training data. The macro averages for precision, recall, and F1-score were 0.97, 0.88, and 0.90, respectively, indicating strong overall performance across the different classes, particularly in precision.

**Figure 9:**
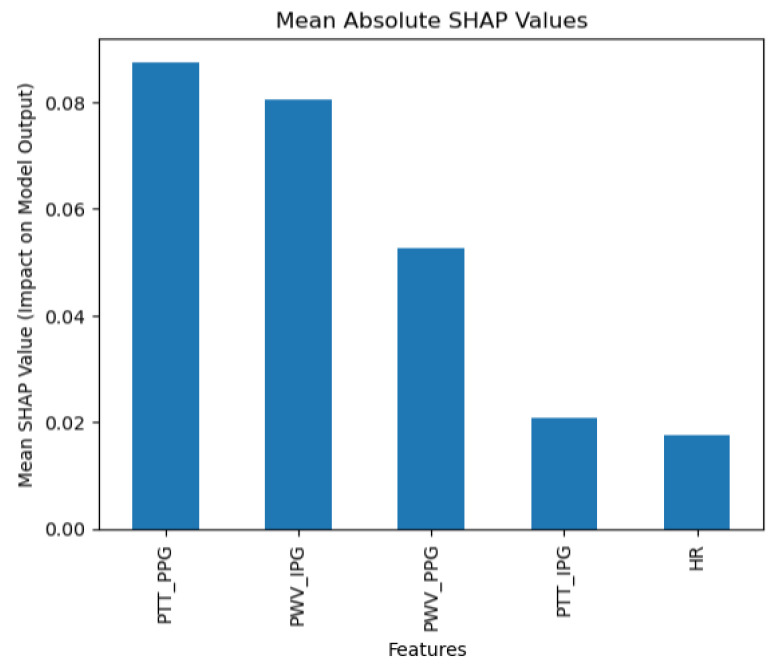
K-Nearest Neighbors SHAP results.

In terms of weighted averages, the precision was 0.94, the recall was 0.93, and the F1-score was 0.92. These values are slightly lower than the macro averages but still demonstrate high efficacy, suggesting that KNN performs well across classes, including those with higher prevalence in the dataset. According to SHAP analysis (Figure 9), the model places significant importance on Pulse Transit Time from Photoplethysmography (PPT_PPG), consistent with other algorithms, highlighting its predictive power.

#### 5.5.5. Naïve Bayes

The Naïve Bayes model achieved an accuracy of 0.86, with confidence intervals for accuracy spanning from 0.67 to 0.89. This range indicates a moderate level of consistency in the model’s predictions across different data subsets. The macro averages—precision of 0.69, recall of 0.75, and F1-score of 0.72—suggest that while Naïve Bayes performs reasonably well across all categories, there is room for improvement, particularly in precision. The weighted averages are slightly higher, with a precision of 0.76, a recall of 0.87, and an F1-score of 0.81. These metrics indicate that the model performs better when adjusted for the prevalence of each class, suggesting that Naïve Bayes is more effective in handling the dominant classes in the dataset but might struggle with minority classes without further tuning.

**Figure 10:**
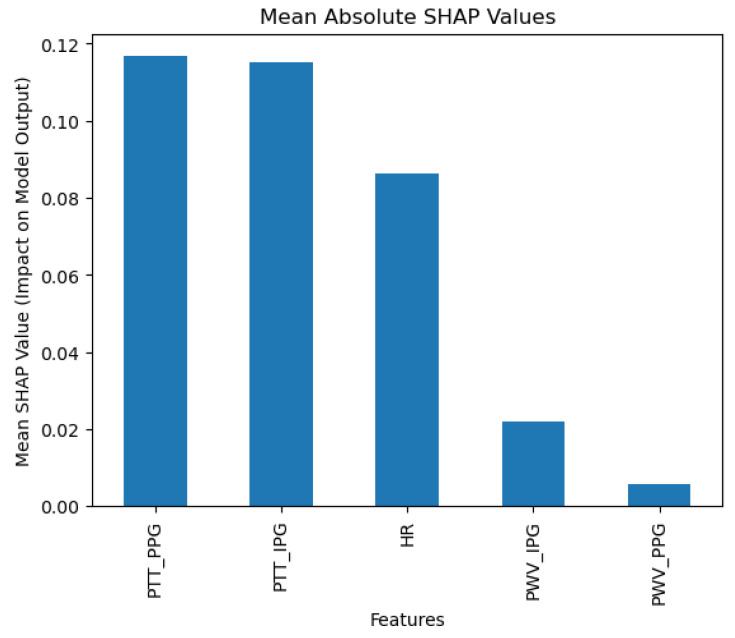
Naïve Bayes SHAP results.

SHAP analysis (Figure 10) emphasizes Pulse Transit Time from Photoplethysmography (PPT_PPG) as a critical feature, aligning with the findings across other models.

Among the models tested, K-Nearest Neighbors demonstrated the highest accuracy (93%) and macro average metrics, while Random Forest showed robustness in handling imbalanced classes. Confidence interval analysis confirmed the reliability of the models across varying datasets. Pulse Transit Time (PPG-derived) emerged as the most critical feature for blood pressure prediction across all models.

## 6. Discussion

In our study, we integrated Impedance Plethysmography (IPG) with Photoplethysmography (PPG) to explore the potential for enhancing blood pressure prediction through non-invasive methods. This combination of modalities aimed to capitalize on the complementary strengths of each technique: IPG’s sensitivity to volumetric blood flow changes and PPG’s ability to accurately track pulse wave dynamics. This approach sought to provide a comprehensive analysis that might overcome some of the limitations faced by current cuffless monitoring technologies, which typically rely on single-signal data acquisition.

Reflecting on similar studies, such as those by Stergiou et al. [15] and Liu et al. [16], our methodology offers a different angle by employing dual-modality data. These studies have validated the efficacy of wearable technologies using primarily electrocardiograms and photoplethysmograms, focusing on single-signal techniques. In contrast, our work attempted to merge the data richness from both IPG and PPG, potentially offering a more detailed physiological snapshot across various states of cardiac activity.

Our analysis employed a diverse array of machine learning algorithms, from ensemble methods like Random Forest to Logistic Regression, Support Vector Machines, K-Nearest Neighbors, and Naïve Bayes. By evaluating metrics such as accuracy, precision, recall, and F1-score, alongside their macro and weighted averages, we gained a comprehensive understanding of each model’s capability to handle both typical and atypical cardiovascular events.

The performance of the machine learning models was assessed across various evaluation metrics, including accuracy, area under the curve (AUC), and macro and weighted averages of precision, recall, and F1-score. These metrics provide a comprehensive understanding of each model’s ability to classify blood pressure levels effectively, and the results highlight significant differences in model performance.

Among the models, K-Nearest Neighbors (KNN) achieved the highest accuracy at 0.93, followed closely by Random Forest at 0.90. Random Forest also recorded the highest AUC of 0.96, indicating its superior ability to differentiate between blood pressure classes. KNN and Naïve Bayes demonstrated strong AUC values of 0.88 and 0.92, respectively, while Support Vector Machine (SVM) and Logistic Regression showed relatively lower AUCs of 0.86 and 0.89.

Random Forest excelled in macro precision (0.95) and macro recall (0.81), showcasing balanced performance across all classes. Its weighted precision, recall, and F1-score also remained consistently high (0.92, 0.90, and 0.88, respectively). KNN performed exceptionally well in macro averages, particularly in macro precision (0.97) and F1-score (0.90), indicating its strong capability in distinguishing between classes. Naïve Bayes showed moderate performance, with slightly lower macro averages but comparable weighted averages.

Confidence intervals for the metrics further confirm the robustness of Random Forest and KNN. Random Forest displayed the narrowest confidence intervals across metrics, such as precision (0.80–0.98), recall (0.81–0.95), and AUC (0.90–1.00), underscoring its stability and reliability. KNN and Naïve Bayes also showed reasonable intervals, although KNN exhibited slightly wider intervals for some metrics, suggesting variability across data splits.

AUC analysis by class revealed that Random Forest consistently outperformed other models, particularly for hypertension stages (AUC = 1.00 for hypertension stages 1 and 2) (Table 5). This indicates its exceptional ability to distinguish between these critical classes. KNN also achieved perfect AUCs for the hypertension stages but struggled with the elevated class (AUC = 0.61). Naïve Bayes demonstrated solid performance across most classes but did not achieve the same level of distinction as Random Forest for critical hypertension categories.

The results underscore Random Forest’s overall superiority, attributed to its ability to handle class imbalances and complex relationships between variables effectively. Its ensemble nature likely contributed to its consistent performance across multiple evaluation metrics and BP ranges. KNN’s high performance, particularly in macro precision and F1-score, can be linked to its distance-weighted approach, which allows it to better classify data points with clear proximity-based patterns. However, KNN’s limitations in handling the elevated class highlight potential issues with unbalanced datasets and subtle class distinctions.

The comparative analysis highlights Random Forest as the most reliable model for non-invasive BP classification, supported by its high accuracy, AUC, and stability across metrics. However, further refinement, particularly for models like Logistic Regression and SVM, could improve their ability to distinguish between blood pressure classes, especially in cases of elevated BP, which remains a challenge across models.

This study highlights the importance of selecting and tuning the right machine learning algorithms based on their ability to accurately detect and send alerts upon abnormal blood pressure levels. An ensemble approach, leveraging the strengths of various models, might offer the most robust solution for developing advanced, reliable, and responsive health monitoring systems. This strategy ensures that users receive timely alerts that could lead to prompt medical interventions, thereby enhancing overall health outcomes.

Moreover, the use of SHAP values in our analysis helped elucidate the contribution of individual features to the models’ predictions, highlighting the significant impact of combining IPG and PPG data. This deeper level of analysis was intended to help pinpoint areas where sensor technology and algorithmic approaches could be refined for better performance.

Despite these contributions, our study is not without its limitations. The dual-modality approach, while offering rich data, also introduces complexity in data processing and analysis. The accuracy of such systems heavily relies on the precision of signal capture and the synchronization between IPG and PPG data streams, which can be challenging to maintain consistently outside of controlled environments. Moreover, our study’s sample was relatively homogeneous and limited to a specific age group, which may affect the generalizability of the findings to other populations or clinical settings. The absence of hypertensive crisis values stems from the inclusion criteria, which focused on healthy individuals aged 18–35 years without chronic conditions or medications affecting blood pressure. Consequently, the dataset predominantly captured normal, elevated, and hypertensive Stage 1 and Stage 2 blood pressure ranges, leaving a critical data gap for the hypertensive crisis category.

This limitation underscores the need for future studies to incorporate a broader population with diverse blood pressure levels, including those in the hypertensive crisis range. Such an approach would enable a more comprehensive evaluation of machine learning models, ensuring their applicability and robustness across the full spectrum of blood pressure conditions. Addressing this limitation will enhance the clinical relevance of these models, particularly in detecting and managing severe hypertension cases.

In comparison to the existing literature, our study adds to the ongoing dialogue about the feasibility and challenges of non-invasive blood pressure monitoring. The integration of IPG and PPG for blood pressure estimation presented here offers a potential pathway for future research, particularly in enhancing the accuracy and reliability of these measurements for broader clinical use. Future work should aim to address the noted limitations by expanding this study’s demographic diversity and conducting tests in more varied real-world environments.

Yavarimanesh et al. evaluated the stability of cuffless BP calibration models over one year, identifying the toe PAT-SBP model as the most reliable, with an RMSE of 9.6 ± 0.8 mmHg and a requirement of annual recalibration [11]. Their study, however, was limited to 10 participants and emphasized long-term recalibration needs [11]. In contrast, our study applied machine learning to classify BP categories in a larger sample of 100 participants, achieving 90% accuracy (Random Forest) with an AUC of 0.96. While Yavarimanesh et al. focused on feature stability over time, our work highlights the robustness of machine learning models for BP classification across pre- and post-exercise conditions. Both studies underscore the importance of feature selection, with toe PAT and PTT PPG emerging as critical predictors.

Zhang et al. demonstrated that pulse arrival time (PAT) was an inadequate surrogate for Pulse Transit Time (PTT) in blood pressure (BP) prediction, with PAT yielding errors ∼80% higher than PTT and failing to meet FDA error limits [12]. PTT was found to be more reliable for BP tracking, particularly during dynamic changes induced by hemodynamic interventions [12].

In contrast, our study focuses on leveraging PTT-based features for everyday BP monitoring in healthy users rather than clinical settings. Using machine learning models, such as Random Forest, we achieved 90% accuracy and a high AUC of 0.96 for BP classification. These results highlight the practicality and robustness of PTT-derived features for non-invasive and cuffless BP monitoring in daily life, aligning with the need for simple and accurate solutions for healthy individuals.

Table 6 compares our study’s findings with key related studies discussed in Section 3.4 (Assessing Machine Learning Algorithms for Blood Pressure Prediction Using PTT), highlighting the focus, performance, limitations, and potential of machine learning in predicting and monitoring blood pressure.

The proposed technology, therefore, should not be viewed as a replacement for traditional blood pressure monitoring devices that provide precise measurements critical for clinical diagnosis and treatment. Instead, it should be seen as a supplementary tool that offers a convenient and non-invasive way to keep a regular check on one’s cardiovascular health, encouraging proactive health management in everyday life settings.

## 7. Conclusions

This study showcases the substantial potential of machine learning algorithms, particularly Random Forest, to leverage Pulse Transit Time (PTT) data from Photoplethysmography and Impedance Plethysmography for approximate blood pressure classification. Our results demonstrate that these models can be effectively integrated into non-invasive continuous health monitoring systems. Such systems are especially beneficial for preventive care by providing real-time alerts for significant deviations in blood pressure that could indicate emerging health issues.

The capacity of these models to consistently monitor and analyze PTT-based readings underscores their potential utility in remote health monitoring settings. These could serve as crucial tools for healthy individuals by alerting them to unusual cardiovascular changes, thus facilitating timely medical consultation or lifestyle adjustments before more serious conditions develop.

Nonetheless, this study is not without its limitations. The dataset used was relatively homogeneous and limited to specific age groups, which might affect the models’ applicability across different demographic profiles. Moreover, challenges such as potential overfitting, particularly with high-performing models like Random Forest, and the handling of class imbalances could impact the stability and reliability of these algorithms in real-world scenarios.

Future research should aim to address these issues by expanding the demographic diversity of study samples and conducting tests in varied real-world environments. Additionally, efforts should be made to refine the algorithms to enhance their ability to manage data imbalances and prevent overfitting, ensuring that the models are robust and reliable for general use.

## Figures and Tables

**Table 1:** Healthy and unhealthy blood pressure ranges used in the analysis [25].

Blood Pressure Category	Systolic mmHg	and/or	Diastolic mmHg
Normal	Less than 120	And	Less than 80
Elevated	120–129	And	Less than 80
High Blood Pressure (Hypertension) Stage 1	130–139	Or	80–89
High Blood Pressure (Hypertension) Stage 2	140 or Higher	Or	90 or Higher
Hypertensive Crisis	Higher than 180	And/Or	Higher than 120

**Table 2:** Demographic information and physiological variables before and after exercise.

Demographics		
Age	24.2 ± 6.34 years	
Sex	50 Female + 50 Male	
BMI	27.5 ± 4.6 kg/m^2^	
**Variables**	**Before Exercise**	**After Exercise**
HR	86.22 ± 12.83 beats/min	112.56 ± 20.00 beats/min
PTT PPG	319.48 ± 36.96 ms	264.16 ± 30.21 ms
PTT IPG	270.78 ± 29.41 ms	208.54 ± 28.49 ms
PWV PPG	4.23 × 10^−3^ ± 4.14 × 10^−4^ m/s	5.12 × 10^−3^ ± 5.56 × 10^−4^ m/s
PWV IPG	4.99 × 10^−3^ ± 5.51 × 10^−4^ m/s	6.53 × 10^−3^ ± 9.52 × 10^−4^ m/s
Blood Pressure Systolic	113.08 ± 8.10 mmHg	141.74 ± 11.40 mmHg
Blood Pressure Diastolic	63.28 ± 6.54 mmHg	70.74 ± 11.34 mmHg
**Blood Pressure Classes**	**Before Exercise**	**After Exercise**
Normal	82	43
Elevated	18	11
Hypertension Stage 1	0	18
Hypertension Stage 2	0	28
Hypertensive	0	0
Total Number	100	100

**Table 3:** Prediction training test results for all classification algorithms.

Classification Algorithm	Accuracy	Area Under the Curve (AUC)	Macro (avg)			Weighted (avg)		
			Precision	Recall	F1-Score	Precision	Recall	F1-Score
Random Forest	0.90	0.96	0.95	0.81	0.82	0.92	0.9	0.88
Logistic Regression	0.70	0.89	0.59	0.54	0.47	0.67	0.70	0.60
Support Vector Machine	0.83	0.86	0.66	0.71	0.68	0.74	0.83	0.78
K-Nearest Neighbors	0.93	0.88	0.97	0.88	0.90	0.94	0.93	0.92
Naïve Bayes	0.86	0.92	0.69	0.75	0.72	0.76	0.87	0.81

**Table 4:** Confidence intervals for performance metrics across various classification algorithms.

Classification Algorithm	95% CI				
	Precision	Recall	F1-Score	Accuracy	AUC
Random Forest	0.80–0.98	0.81–0.95	0.77–0.95	0.80–0.95	0.90–1.00
Logistic Regression	0.64–0.82	0.72–0.84	0.64–0.81	0.72–0.84	0.83–0.94
Support Vector Machine	0.68–0.81	0.71–0.89	0.65–0.84	0.71–0.89	0.83–0.93
K-Nearest Neighbors	0.63–0.86	0.63–0.87	0.60–0.85	0.63–0.87	0.82–0.97
Naïve Bayes	0.65–0.83	0.67–0.89	0.64–0.85	0.67–0.89	0.83–0.97

**Table 5:** AUC values by class for each machine learning model.

AUC for Each Class	Random Forest	Logistic Regression	Support Vector Machine	K-Nearest Neighbors	Naïve Bayes
Normal (0)	0.99	0.93	0.92	0.93	0.94
Elevated (1)	0.81	0.73	0.73	0.61	0.75
Hypertension 1 (2)	1.00	0.99	0.83	1.00	1.00
Hypertension 2 (3)	1.00	0.95	0.96	1.00	1.00

**Table 6:** Comparative summary of machine learning studies on blood pressure prediction and management.

Aspect	This Study	Krittanawong et al. [22]	Layton [21]	Mroz et al. [19]	Montagna et al. [18]
**Focus**	Predictive model for BP to be used in wearables.	AI for hypertension prediction and care.	ML in hypertension diagnosis/treatment.	Personalized BP management via AI.	Integrating diverse BP data sources.
**Best ML Model**	KNN and Random Forest (RF).	RF and SVM.	Neural networks and SVM.	Decision Trees and Adaptive Learning.	Clustering and DNNs.
**Accuracy**	KNN: 93% and RF: 90%.	~90%.	~85–90%.	Not specified and adaptive-focused.	~85% precision, which varies with data.
**AUC**	KNN: 0.88 and RF: 0.96.	~0.85–0.90.	~0.85–0.90.	Not specified, with the trial being design-focused.	~0.80–0.88.
**Other** **Metrics**	Precision: KNN 0.97 and RF 0.95; Recall: KNN 0.88.	Limited precision and recall analysis.	Broader metrics, with no precision details.	General trends, with no breakdowns.	Focuses on trends, with no macro averages.
**Robustness**	Highly consistent metrics across models.	Moderate precision and limited generalizability.	Moderate precision, with interpretability issues.	Strong precision and reliance on multi-source data.	Moderate precision, with integration challenges.
**Limitations**	Bias in wearable data, with limited generalizability.	Lacks real-world validation.	Neural network interpretability issues.	Requires high-quality and multi-source data.	Data integration, with real-world gaps.

## Data Availability

Data supporting the results reported in this study are available upon reasonable request. Due to privacy and ethical considerations, the dataset generated and analyzed during this study is not publicly available. Requests for access to the data can be directed to the corresponding author and will be evaluated in accordance with institutional and ethical guidelines.
